# Editorial: Novel biomarkers in tumor immunity and immunotherapy

**DOI:** 10.3389/fimmu.2024.1405082

**Published:** 2024-04-08

**Authors:** Takaji Matsutani, Esra Akbay, Eyad Elkord

**Affiliations:** ^1^Translational Research Department, Drug Development Laboratories, Kyoto R&D Center, Maruho Co., Ltd., Kyoto, Japan; ^2^Department of Pathology, University of Texas Southwestern Medical Center, Dallas, TX, United States; ^3^Department of Biological Sciences, School of Science, Xi’an Jiaotong-Liverpool University, Suzhou, Jiangsu, China; ^4^Biomedical Research Center, School of Science, Engineering and Environment, University of Salford, Manchester, United Kingdom

**Keywords:** biomarker, ICI, bioinformatics, tumor immunology, transcriptome, microbiome

In this Research Topic, numerous researchers reported novel biomarkers and methodologies for predicting the efficacy of cancer immunotherapy across various cancers. Additionally, a wide spectrum of fundamental research has been conducted, leading to the discovery of biomarkers. Alongside traditional immunological analyses, a diverse array of methodologies such as bulk RNA-Seq, scRNA-Seq, and bacterial flora analysis have been employed. Moreover, state-of-the-art bioinformatics technologies have been effectively utilized in biomarker discovery. These investigations not only unveil intriguing new discoveries facilitated by cutting-edge technologies but also hold significant promise for shaping the future landscape of tumor immunology.

We are pleased to present this successful Research Topic to the scientific community. This Research Topic comprises six reviews and forty-one original papers. Four systemic reviews on predicting response to immune checkpoint inhibitors (ICI) were published: Qian et al. conducted a meta-analysis, affirming that plasma EBV DNA levels serve as reliable biomarkers for predicting favorable responses to ICI treatment in nasopharyngeal cancer patients, Rugambwa et al. established an association between high neutrophil-lymphocyte and platelet-lymphocyte ratios and poorer ICI treatment outcomes, and Fejza et al. presented accumulating evidence indicating extracellular matrix molecules as biomarkers identifying patients benefiting from ICI treatment. Shi et al. compared various predictive biomarker testing methods for ICI efficacy, while Wu et al. reviewed small molecule inhibitors for KRAS mutant cancers. Fonseca-Montaño et al. delved into the significance of long-non coding RNAs (lncRNAs) in breast cancer and their latest findings. These reviews furnish insights into the current status of previous studies in the realm of tumor immunology, aiding in the recognition and anticipation of forthcoming challenges.

Biomarkers encompass cancer-specific and cancer-nonspecific markers applicable across diverse cancer types. Within this purview, several intriguing factors have been identified as treatment response and prognosis markers in pan-cancer patients. Dong et al. revealed the multifaceted role of Proteasome Activator Complex Subunit 3 (PSME3) in tumors, establishing it as a pan-cancer prognostic marker. Lin et al. proposed Glioma pathogenesis related-2 (GLIPR2) as a promising novel biomarker and tumor suppressor. Liu et al. examined the functional attributes of Tubulin epsilon and delta complex 2 (TEDC2) in human tumors, identifying TEDC2 as a prognostic marker across various tumor types. Li et al. elucidated the role of disulfidoptosis-related genes (DRGs) in pan-cancer prognosis and their interplay with immunity, constructing a prognostic model utilizing various bioinformatics and machine learning techniques. Zhu et al. highlighted that high expression of Origin recognition complex 6 (ORC6) could serve as a prognostic biomarker in pan-cancer patients. Wei et al. showed the positive correlation between elevated expression levels of IFN-γ-related genes and drug sensitivity, emphasizing the pivotal role of IFN-γ in tumor immunotherapy. Pan et al. reported on the involvement of integrin-binding sialic acid protein (IBSP), a member of the small integrin-binding ligand N-linked glycoprotein (SIBLING) family, in tumorigenesis across various cancers, proposing IBSPs as prognostic biomarkers and immunotherapy targets in pan-cancer. Wu et al. delineated the prognostic potential of the Ferroptosis-related gene Hypermethylated in Cancer 1 (HIC1) in various cancers, indicative of its utility in predicting cancer prognosis, immunotherapy response, and drug sensitivity. Li et al. demonstrated the significant correlation of Thymosin beta-10 (TMSB10) with the tumor microenvironment and immune regulatory factors, advocating its role as a predictive marker for therapeutic response in cancer patients. Huang et al. identified Four Jointed Box 1 (FJX1) as a novel prognostic factor crucial in tumor immunity based on comparative expression profile analysis. Sun et al. established an association between dysregulation of the proprotein convertase subtilisin/kexin-9 (PCSK9) and poor clinical outcomes, suggesting its potential as a robust pan-cancer biomarker. These studies link these genes previously not directly linked to oncogenesis or tumor immunity to immune regulation and suggest potential role as biomarkers.

Studies focusing on specific tumors have unveiled several therapeutic and prognostic markers in hepatocellular carcinoma (HCC). Shi et al. developed the PCD Index (PCDI), comprising programmed cell death-related genes, as a prognostic and treatment response predictor in HCC. Zhang et al. observed elevated expression of DnaJ heat shock protein family member C8 (DNAJC8) in HCC tissues, correlating with poor prognosis and demonstrating its oncogenic role. Jiang et al. identified a significant correlation between CD93 expression and the prognosis of liver hepatocellular carcinoma patients. Xu et al. elucidated abnormal T follicular helper cell infiltration associated with forkhead box M1 (FOXM1) as a crucial prognostic factor in HCC patients.

Prominent biomarkers have also emerged from studies on lung adenocarcinoma (LUAD) and lung squamous cell carcinoma (LUSC). Li et al. focused on coagulation- and macrophage-associated (COMAR) genes, constructing a COMAR risk score model predictive of prognosis and clinical outcome in LUAD patients. Zhu et al. identified twelve HUB genes via Weighted Gene Coexpression Network Analysis (WGCNA), potentially implicated in LUAD progression via immune-related signaling pathways. Wu et al. derived LUSC-specific differentially expressed gene signatures (7-DEGs) with prognostic significance for LUSC patients.

A multitude of original and intensive investigations have explored valid biomarkers across a diverse array of tumors. Li et al. identified hub biomarkers closely associated with gastric cancer (GC) using microarray data and algorithmic approaches. Cai et al. delineated the multifaceted role of Fibroblast activation protein (FAP) in gastrointestinal cancer progression. Deng et al. developed a prognostic panel using hypoxia-related genes, predicting clinical prognosis and treatment efficacy in GC. Chen et al. devised a prognostic score model based on tumor microenvironment (TME)-related genes, effectively predicting breast cancer patient prognosis and chemotherapy efficacy. Wei et al. employed immune- and cancer-associated fibroblast (CAF)-associated genes (ICRGs) to prognosticate and evaluate immunotherapy efficacy in colorectal adenocarcinoma patients. Hailang et al. identified the gene encoding mitochondrial Aspartyl-tRNA synthetase 2 (DARS2) as a prognostic biomarker in bladder cancer. Dong et al. unveiled the impact of necroptosis-associated myeloid lineages on the immune landscape of pancreatic cancer through scRNA-Seq analysis. Liu et al. conducted LASSO and Cox regression analyses on angiogenesis-related genes (ARGs) in soft-tissue sarcomas (STS) to establish a novel ARG signature (ARSig). Their study demonstrated that ARSig holds promise as an independent prognostic predictor for STS. Li et al. demonstrated that C15orf48, an inflammatory response-related gene, could be a potential biomarker for tumor prognosis and a target for immunotherapy in thyroid cancer. Jiang et al. identified two immunogenic cell death (ICD) subtypes through consensus clustering analysis and constructed an ICD prognostic signature capable of predicting overall survival in patients with renal clear cell carcinoma.

Recent insights underscore the pivotal role of the gut microbiota in the cancer microenvironment and its influence on the efficacy of immunotherapies such as ICIs. Multiple studies have been dedicated to this research area. Zhao et al. reported that enrichment of the gut microbiota, particularly Lachnoclostridium, correlates with the presence of intratumoral tertiary lymphoid structures (TLS) in HCC patients. Gorgulho et al. proposed an immune-microbial score comprising the relative abundance of CD3+HLADR+, NLR, and enterobacteria, which demonstrated predictive capability for therapeutic response to ICIs. Hamada et al. identified bacteria implicated in the efficacy of ICIs and immune-related adverse events (irAEs), suggesting promise for developing a marker to predict cancer immunotherapy efficacy through gut microbiota and fecal transplantation applications.

Several novel and useful biomarkers have emerged from serological methods. Hou et al. identified serum cytokines and the neutrophil-to-lymphocyte ratio as effective biomarkers for predicting the efficacy of ICIs in gastric cancer. Liu et al. introduced an inflammatory prognostic index (InPI) based on three inflammatory markers in patients with relapsed/refractory multiple myeloma (R/R MM) treated with CAR-T therapy, demonstrating its validity as a prognostic biomarker. Raza et al. identified novel immunosuppressive/stimulatory soluble mediators as surrogate and predictive biomarkers of tissue PD-L1 (TPD-L1) status, treatment response, and progression-free survival (PFS) in NSCLC patients treated with ICI.

Many studies have shown that the development of new methods and a multifaceted approach can help in the development of new biomarkers. Ohkuma et al. developed a highly sensitive quantitative immunohistochemical method employing phosphor-integrated dots (PID) for evaluating PD-L1 expression quantitatively. Utilizing this method, they were able to detect PD-L1 expression in the tumors of a subgroup of patients with a favorable prognosis with ICI. Zhang et al. established an alternative splicing (AS) prognostic signature based on AS subtypes in clear cell carcinoma (ccRCC), emphasizing the importance of the AS-SF network, inclusive of splicing factors (SFs), in studying regulatory mechanisms. Yang et al. introduced the CRP-albumin-lymphocyte (CALLY) index, which combines C-reactive protein (CRP), albumin, and lymphocytes, demonstrating its superior prognostic value compared to classical prognostic factors in colorectal cancer patients. Liu et al. introduced a novel biomarker for breast cancer, a nectin-4-specific scFv, with diagnostic and therapeutic applications, recognizing nectin-4 expressed by breast cancer cells *in vitro* and *ex vivo*. Zhou et al. identified CD26lowPD-1+ CD8 T cells associated with acute myeloid leukemia (AML) progression and described the prognostic significance of CD26 in AML. Inaba et al. suggested amino acid polymorphisms of HLA class II molecules and HLA-DP5 as genetic predictors of ICI-T1DM in type 1 diabetes induced by ICIs. Wang et al. demonstrated the utility of 18F-fluorodeoxyglucose positron emission tomography (18F-FDG PET) as an imaging biomarker for predicting pathologic response and prognosis in patients with unresectable hepatocellular carcinoma treated with lenvatinib and PD-1 as a conversion therapy.

The compilation of studies in this Research Topic explores various facets of tumor immunology, focusing on identifying novel biomarkers and predictive methods for cancer immunotherapy across diverse cancer types. Researchers employ advanced technologies to uncover promising biomarkers with implications for treatment response and prognosis in cancer patients. Systematic reviews and original papers shed light on the multifaceted landscape of tumor immunology, exploring biomarkers ranging from traditional immunological markers to emerging candidate biomarkers. Notably, investigations extend beyond cancer-specific markers, revealing the involvement of interesting molecules in cancer progression. Moreover, studies elucidate the role of the gut microbiota in modulating the tumor microenvironment and response to immunotherapy, offering insights into potential therapeutic interventions. Serological methods offer valuable biomarkers, while technological advancements, including quantitative immunohistochemical methods, enhance prognostic accuracy. This comprehensive body of work not only highlights the current state of tumor immunology research but also paves the way for future advancements in cancer diagnosis, prognosis, and treatment.

## Author contributions

TM: Writing – original draft, Writing – review & editing. EA: Writing – review & editing. EE: Writing – review & editing.

